# Evaluation and Comparison of the Nutritional and Mineral Content of Milk Formula in the Saudi Arabia Market

**DOI:** 10.3389/fnut.2022.851229

**Published:** 2022-06-09

**Authors:** Nora A. Alfaris, Zeid A. Alothman, Tahany S. Aldayel, Saikh M. Wabaidur, Jozaa Z. Altamimi

**Affiliations:** ^1^Department of Physical Sport Science, College of Education, Princess Nourah bint Abdulrahman University, Riyadh, Saudi Arabia; ^2^Department of Chemistry, College of Sciences, King Saud University, Riyadh, Saudi Arabia

**Keywords:** infant formula, protein, fat, lactose, total solids

## Abstract

**Background/Aim:**

As recommended by WHO, breastfeeding is the best choice and safe for infants. The formula for infants plays an imperative role in the infant's diet and remains an excellent alternative for breast milk. The milk formula for most infants has been increasingly changed with various compositions to create a similar breast milk production. This study aims to analyze and determine the chemical composition of a few milk formulas available in the Saudi Arabian market.

**Materials and Methods:**

Thirty-five milk formula samples for infants of different age categories were collected from Riyadh City and analyzed for protein, fat, carbohydrates, lactose, total solids, total non-fat solids, calcium, iron, and zinc. Among batches collected, there were 15 branded products suitable for those of age 0–6 months, five for those of age 0–12 months, four for those of age 1–3 years, and 11 for those of age 6–12 months.

**Results:**

For infants, the milk formula sample parameters investigated varied significantly (*p* ≤ 0.05). A significantly high protein value was 22.72% for a brand for infants with an age of 0–6 months, and the lowest was 11.31% for a brand for those of age 0–12 months. Fat content was high in a brand (26.92%) for infants of age 0–6 months and low in a brand (17.31%) for those aged 6–12 months. The high value of carbohydrates was found in a brand (60.64%) for those of age 0–6 months and a low one (44.97%) in a brand for those of age 0–12 months. The total energy, lactose, total solids, total non-fat solids, and minerals (calcium, iron, zinc) were significantly (*p* ≤ 0.05) varied between milk formulas at the same age.

**Conclusion:**

There were significant variations between milk formulas of the same ages. According to age groups, some nutrients were not identical to the reference values for children's food.

## Introduction

Milk formulas are the only food that plays an important role in the nutrition of non-breastfed infants and premature infants. It provides them with all the nutrients needed for healthy growth and is a mixture of fats, proteins, and carbohydrates (1). According to the WHO, breastfeeding is the best nutrition, but infant formula has an important role in the infant's diet and remains a substitute for breast milk (2). Once breastfeeding is not possible for infants, formula milk is normally used as a substitute for human breastmilk and performs an indispensable role in infants' growth, providing proper nutrition (3). In addition to breast milk, milk formulas are the only alternative product considered nutritionally acceptable by the medical authority for infants <1 year of age (4).

The most commonly used milk formulas, depending on the manufacturer, contain a source of protein such as pure cow's milk, whey, and casein, a source of fat such as a blend of vegetable oils, a source of carbohydrates such as lactose, and a blend of vitamins, minerals, and other ingredients. However, soy is used in some milk formulas as a source of protein in place of cow's milk, and others use reduced (hydrolyzed) protein for infants who are allergic to other proteins (5). It is critical to ensure that infant formula feeding aims to approximate the nutritional characteristics of human milk rather than mimicking human milk, which is qualitatively incomparable. According to Fanaro et al. (6), milk formulas with a protein content of 2–2.5 g/100 ml and a protein/energy ratio of <3 g/100 kcal are used for normal infants, while with higher protein content (2.9 g/100 ml) and a higher protein/energy ratio (3.5 g/100 kcal) are for very low birth weight or preterm infants.

A study conducted by Michaelsen and Greer (7) showed that high protein content in milk formula is associated with excess weight gain in infancy, leading to a 20% risk of obesity later in life. Hernell (8) reported that the composition of a milk formula has been improved, not only to increase nutritional similarities with human milk but also to include ingredients with additional health benefits for infants. Proteins (-lactalbumin and lactoferrin), milk fat globule membrane, taurine, folates, polyamines, polyunsaturated fatty acids, prebiotics, and probiotics are among these ingredients. Although lactose is the leading carbohydrate source in milk formula, up to 30% of total non-lactose carbohydrates, such as maltodextrins, can be added (8).

The factors such as product composition (mainly carbohydrate, fat, and protein), storage conditions, handling of the products, and transportation conditions, such as relative humidity and temperature, were reported to affect the physicochemical properties of milk formulas (9). The milk formula is commonly spray-dried into a powdered form to ensure longer shelf life and facilitate handling, storage, and transportation (10). The selection of suitable milk formula should be based on the infant's requirements and medical advice (11) to ensure that the product is comparable to breast milk (12). The availability of different types of milk formulas in the market confused both parents and physicians and made it more challenging to choose the best formula for infants (13).

In addition, advertising of milk formula is not only in the form of promotion directed to the physician but also in illegal forms, such as direct-to-consumer advertising (mothers), which is permitted in developing countries (14), which may further complicate the scientific decision (15) to choose the best formula milk. The basic composition of milk formula has increasingly been changed to produce a product with utmost similarity with breast milk. The present study was carried out to evaluate and compare the nutritional quality of infant formula milk available in the Saudi Arabian market.

## Materials and Methods

### Materials

Thirty-five different brands of milk formulas were obtained from pharmacies in Riyadh, Saudi Arabia. The samples were chosen based on market availability and usage. There were 15 branded products suitable for those aged 0–6 months, five for those aged 0–12 months, 4 for those aged 1–3 years, and 11 for those aged 6–12 months among the batches collected. Before analysis, the samples were kept at room temperature. The composition of all formulas was meticulously gathered from the information written on the formulas' containers. During analysis, the samples were kept in a desiccator to keep the environment sealed. All chemicals used in this study were of reagent grade.

### Protein Determination

The protein content was determined by the formal titration method according to AOAC (16). The crude protein content of the milk formulas was calculated by multiplying nitrogen content by the factor for dairy products of 6.25.

### Fat Determination

Gerber's method was used for the determination of the fat content of milk formula samples. Exactly 10 ml of H_2_SO_4_ and 11 ml of milk samples were taken in a 22-ml capacity butyrometer. The mixture was shaken thoroughly. After that, 1 ml of amyl alcohol was added to develop three clear layers. A cork was inserted into the mixture. Then, the mixture was centrifuged at 1,100 rpm for 4 min. After centrifugation, the test bottles were heated in a water bath at 60°C. Finally, the fat was separated at the neck of the butyrometer and measured directly through the main division of the scale (17).

### Carbohydrate Determination

To determine the carbohydrates of milk formula samples, the sum of the values of protein, fat, moisture, and ash as a percent (w/w) was subtracted from 100.

### Total Energy Determination

Atwater factors for protein, carbohydrate, and fat were applied to determine the total energy. The general values (four caloric for 1-g protein, four caloric for 1-g carbohydrate, nine caloric for 1-g fat) were used. Calories were calculated following the equation:


$$\begin{array}{l} \left(4\times \text{protein}\right)+\left(4\times \text{carbohydrate}\right)+\left(9\times \text{fat}\right)\\ =\text{caloric content per 100 g or ml}. \end{array}$$

### Total Solids Determination

An oven-dried method was applied to determine the total solid contents of the milk formula samples. A fresh formula was taken and placed in a pre-weighed dish. Then, the content evaporated in the steam bath. After that, the dish content was dried in an oven at 101°C till constant weight was obtained. The dried samples were placed in a desiccator, kept for 1 h in the presence of silica gel, and weighed. The total solids percent was determined following the formula according to AOAC (16).


$$\begin{array}{l} \text{Total solid}\;\%=\text{Weight of a dried sample}/\text{Weight of}\\ \text{a milk sample}\times \;100. \end{array}$$

### Total Non-Fat Solids Determination

Percent total non-fat solids were calculated according to the Bassbasi et al. (18) method. The solids-non-fat content was determined after discarding fat from milk and weighing the residue.


Total non-fat solids=Total solid-fat.

### Lactose Determination

The lactose of the milk formula was determined using the colorimetric method as described by Abu-Lehia, (19). Briefly, 5 ml of trichloroacetic acid was added to the 5-ml formula and mixed gently. The mixture was filtered using filter paper (Whatman no. 40). Then, 2 ml of the filtrate was diluted to 100 ml with distilled water. The diluted filtrate (2.5 ml) was placed in a 15-ml tube fitted with a screw cap and mixed thoroughly. The content was immersed in a boiling water bath for 2.5 min. Then, the tube was cooled immediately under cold tap water, and distilled water (7 ml) was added to the mixture and mixed thoroughly. Finally, the absorbance was measured at 520 nm. A standard curve was prepared by plotting the absorbance against the corresponding lactose concentrations.

### Analysis of Minerals

The analysis of minerals (calcium, iron, and zinc) was done using atomic absorption spectrometry (Z-2000, Hitachi, Japan) as described by the standard method of GB 5413.21 (20). Each sample was analyzed in triplicate.

### Statistical Analysis

The analysis of each sample was performed in triplicate. The data were analyzed using SPSS statistical software (version 25, IBM Corp., Melbourne, Australia). The data obtained were presented as the mean ± SD. To determine the significant difference among the mean values at *p* ≤ 0.05, a one-way ANOVA was performed.

## Results and Discussion

### Macronutrients and Total Energy of Infant Formulas

Table 1 shows the chemical composition of milk formulas for an infant aged 0 to 6 months. The protein content of milk formulas was varied, with a significantly (*p* ≤ 0.05) highest value of 22.72% for Brand 706, followed by Brand 606 with a value of 14.11%, and the least value of 10.42% for Brand 1,006. As shown in Table 2, the protein content of the formula developed for those of age 0–12 months also varies among brands, with Brand 4,012 having a significantly (*p* ≤ 0.05) high protein content (19.52%) and Brand 2,012 having the least value (11.31%). Moreover, the brands for those of age 6–12 months, Brand 7,612, had a significantly (*p* ≤ .05) higher protein content (20.91%) than other brands, and the Brand 10,612 had the least content (12.81%). However, among the formulas for those of age 1–3 years (Table 3), a significantly (*p* ≤ 0.05) highest protein content was recorded for Brand 413 (18.71%) and least value for Brand 313 (13.12%).

**Table 1:** The chemical composition (%) of milk formulated for an infant with an age ranging from 0 to 6 months.

**Brand**
**No**.	**Protein (%)**	**Fat (%)**	**CHO (%)**	**Energy (Kcal/100 g)**	**Lactose (%)**	**Total solids (%)**	**Total non-fat solids (%)**	**Ca (mg/100 g)**	**Fe (mg/100 g)**	**Zn (mg/100 g)**
106	12.51^d^ ± 0.24	26.61^a^ ± 0.23	50.07^i^ ± 0.36	489.81^e^ ± 1.34	50.11^g^ ± 0.57	97.81^e^ ± 1.04	72.01^h^ ± 0.76	417.91^d^ ± 1.83	5.62^d^ ± 0.14	3.96^c^ ± 0.21
206	12.61^d^ ± 0.78	26.92^a^ ± 0.61	51.55^h^ ± 0.41	498.92^b^ ± 1.32^a^	51.62^g^ ± 0.59	99.41^c^ ± 1.34	73.51^g^ ± 0.84	382.52^f^ ± 2.04	4.57^e^ ± 0.47	4.27^b^ ± 0.52
306	10.70^a^ ± 0.34	22.81^e^ ± 0.46	57.78^c^ ± 0.74	479.21^i^ ± 2.04	57.81^c^ ± 0.66	98.82^d^ ± 1.46	77.72^d^ ± 1.02	377.63^g^ ± 1.66	0.10^g^ ± 0.01	0.43^e^ ± 0.02
406	12.91^d^ ± 0.57	18.53^a^ ± 0.57	58.53^b^ ± 0.82	452.53^m^ ± 2.24	58.61^b^ ± 1.04	97.22^e^ ± 1.31	80.91^b^ ± 1.11	378.31^g^ ± 1.45	3.64^f^ ± 0.51	2.80^d^ ± 0.33
506	12.62^d^ ± 0.44	25.82^b^ ± 0.74	58.64^b^ ± 0.85	517.44^n^ ± 3.21^a^	58.71^a^ ± 1.11	104.31^a^ ± 2.94	80.72^b^ ± 0.77	368.62^h^ ± 2.46	7.37^b^ ± 0.62	4.42^b^ ± 0.57
606	14.11^b^ ± 0.91	20.81^f^ ± 0.68	53.87^f^ ± 0.91	459.22^k^ ± 2.04	53.92^f^ ± 1.21	96.52^f^ ± 1.61	77.51^d^ ± 0.89	367.91^h^ ± 3.24	5.72^d^ ± 0.72	3.27^c^ ± 0.44
706	22.72^a^ ± 0.79	19.01^g^ ± 0.58	48.76^j^ ± 0.45	457.01^l^ ± 3.67	48.83^h^ ± 1.24	100.11^b^ ± 1.88	81.61^a^ ± 1.06	754.12^a^ ± 2.54	11.73^a^ ± 0.93	13.97^a^ ± 0.83
806	11.83^d^ ± 0.45	22.21^e^ ± 0.62	58.15^b^ ± 0.51	479.82^i^ ± 4.01	58.22^b^ ± 1.24	99.42^c^ ± 1.13	79.32^c^ ± 1.44	362.13^i^ ± 2.21	7.54^b^ ± 0.68	2.72^d^ ± 0.66
906	11.32^e^ ± 0.47	24.23^c^ ± 0.49	55.72^a^ ± 0.39	486.23^f^ ± 2.57	55.82^d^ ± 1.05	98.81^d^ ± 0.99	76.42^e^ ± 0.88	424.81^c^ ± 3.01	4.72^e^ ± 0.51	3.15^c^ ± 0.57
1,006	10.42^f^ ± 0.71	22.12^e^ ± 0.48	60.64^a^ ± 0.41	483.34^h^ ± 2.84	60.72^a^ ± 1.01	100.11^b^ ± 2.79	80.42^b^ ± 1.07	481.12^b^ ± 2.49	4.21^e^ ± 0.53	2.71^d^ ± 0.43
1,106	11.41^d^ ± 0.24	24.91^c^ ± 0.51	55.87^d^ ± 0.37	493.32^c^ ± 3.66	55.91^d^ ± 1.07	99.82^a^ ± 1.67	76.62^e^ ± 0.79	417.81^d^ ± 1.24	5.79^d^ ± 0.61	3.21^c^ ± 0.53
1,206	13.32^c^ ± 0.29	22.52^a^ ± 0.68	52.34^g^ ± 0.49	465.31^j^ ± 2.76	52.42^f^ ± 0.94	96.61^f^ ± 1.68	75.12^f^ ± 0.79	334.92^k^ ± 3.29	6.03^c^ ± 0.65	2.68^d^ ± 0.63
1,306	12.43^d^ ± 0.49	25.42^b^ ± 0.55	51.73^h^ ± 0.55	485.43^g^ ± 1.98	51.81^a^ ± 0.84	97.91^e^ ± 1.04	73.51^g^ ± 0.59	360.63^j^ ± 4.01	6.56^c^ ± 0.72	3.93^c^ ± 0.56
1,406	12.22^d^ ± 0.66	23.21^d^ ± 0.69	58.36^b^ ± 0.62	491.21^d^ ± 2.01	58.42^b^ ± 1.04	100.61^b^ ± 2.04	80.01^b^ ± 1.01	405.01^e^ ± 3.33	7.13^b^ ± 0.83	4.21^b^ ± 0.72
1,506	12.71^d^ ± 0.52	24.00^c^ ± 0.45	54.90^e^ ± 0.44	486.45^f^ ± 2.78	54.91^e^ ± 1.12	99.52^c^ ± 1.22	77.01^d^ ± 1.41	303.42^l^ ± 1.89	0.55^g^ ± 0.08	0.42^e^ ± 0.03

*Values are means ± SD of three replicates. Mean values in a column with different superscripts (a, b, c) are significantly different at level p ≤ 0.05. CHO, carbohydrates. Brand No.: 1^st^ digit (1, 2, 3, …, 14, 15) is a serial number; digits 06 denote age 0–6 months*.

**Table 2:** Chemical composition (%) of milk formula for an infant with age ranging from 0 to 12 months and from 6 to 12 years.

**Brand**
**No**.	**Protein (%)**	**Fat (%)**	**CHO (%)**	**Energy (Kcal/100 g)**	**Lactose (%)**	**Total solids (%)**	**Total non-fat solids (%)**	**Ca (mg/100 g)**	**Fe (mg/100 g)**	**Zn (mg/100 g)**
**0–12 months**										
1,012	11.51^d^ ± 0.35	23.41^c^ ± 0.89	44.97^e^ ± 0.41	436.61^d^ ± 1.34	45.01^e^ ± 1.24	88.81^e^ ± 1.12	65.71^e^ ± 0.84	430.81^e^ ± 2.14	0.03^c^ ± 0.01	0.43^b^ ± 0.12
2,012	11.31^d^ ± 0.66	24.13^b^ ± 0.79	49.73^c^ ± 0.57	461.32^c^ ± 1.69	49.81^c^ ± 1.24	93.82^c^ ± 0.94	70.42^d^ ± 0.68	436.42^d^ ± 3.11	0.52^b^ ± 0.04	0.38^b^ ± 0.04
3,012	12.73^c^ ± 0.59	18.41^a^ ± 0.55	51.95^b^ ± 0.91	424.43^e^ ± 2.25	52.02^b^ ± 1.24	91.21^d^ ± 0.88	74.01^c^ ± 0.77	494.61^b^ ± 2.72	0.03^c^ ± 0.01	0.43^b^ ± 0.06
4,012	19.52^a^ ± 0.45	23.82^c^ ± 0.47	48.64^d^ ± 0.58	487.01^b^ ± 3.44	48.71^d^ ± 1.24	101.21^b^ ± 1.14	78.02^b^ ± 0.59	664.93^a^ ± 4.04	11.97^a^ ± 0.94	3.99^a^ ± 0.64
5,012	13.61^b^ ± 0.61	26.31^a^ ± 0.72	63.37^a^ ± 0.47	544.71^a^ ± 2.35	63.42^a^ ± 1.24	109.62^a^ ± 1.12	86.51^a^ ± 0.48	371.81^c^ ± 2.45	0.06^c^ ± 0.02	0.40^b^ ± 0.01
**6–12 months**										
1,612	13.71^g^ ± 0.52	19.11^d^ ± 0.67	62.37^b^ ± 0.49	476.31^d^ ± 1.79	62.41^b^ ± 0.57	102.41^a^ ± 1.01	85.62^b^ ± 0.89	477.11^g^ ± 1.81	0.57^f^ ± 0.05	0.45^e^ ± 0.04
2,612	15.42^e^ ± 0.67	17.52^e^ ± 0.46	57.54^d^ ± 0.69	449.52^a^ ± 2.46	57.62^c^ ± 0.69	98.52^e^ ± 0.91	82.72^e^ ± 0.92	718.92^e^ ± 1.34	0.07^f^ ± 0.08	0.45^e^ ± 0.12
3,612	14.92^f^ ± 0.72	18.91^d^ ± 0.59	55.56^e^ ± 0.83	452.12^g^ ± 1.88	55.61^d^ ± 0.39	97.41^f^ ± 0.84	80.01^f^ ± 0.95	747.91^d^ ± 2.34	6.65^d^ ± 0.29	3.32^c^ ± 0.68
4,612	16.61^d^ ± 0.81	18.41^d^ ± 0.69	58.47^c^ ± 0.75	466.01^e^ ± 2.21	58.52^b^ ± 0.93	101.32^b^ ± 1.02	84.81^c^ ± 0.57	278.43^k^ ± 1.94	7.42^c^ ± 0.31	4.33^b^ ± 0.74
5,612	18.51^c^ ± 0.58	24.92^a^ ± 0.44	49.75^g^ ± 0.59	497.31^a^ ± 3.04	49.81^f^ ± 0.69	102.34^a^ ± 1.12	78.12^g^ ± 0.63	336.01^j^ ± 1.57	9.77^a^ ± 0.83	5.50^a^ ± 0.49
6,612	20.33^a^ ± 0.79	17.91^e^ ± 0.36	53.05^f^ ± 0.62	454.72^g^ ± 1.79	53.12^e^ ± 0.72	100.13^c^ ± 1.04	83.41^d^ ± 0.63	809.51^b^ ± 1.72	8.36^b^ ± 0.69	4.44^b^ ± 0.71
7,612	20.91^a^ ± 0.46	21.93^c^ ± 0.68	46.03^i^ ± 0.39	465.12^ef^ ± 3.03	46.13^h^ ± 0.57	98.81^e^ ± 0.99	76.93^h^ ± 0.39	943.64^a^ ± 3.04	7.31^c^ ± 0.73	5.17^a^ ± 0.67
8,612	13.91^g^ ± 0.66	17.31^e^ ± 0.57	63.27^a^ ± 0.48	464.51^f^ ± 2.82	63.32^a^ ± 0.88	101.12^b^ ± 1.01	86.61^a^ ± 0.48	464.13^h^ ± 1.23	8.64^b^ ± 0.58	2.88^d^ ± 0.51
9,612	14.62^f^ ± 0.57	18.01^d^ ± 0.55	58.26^c^ ± 0.71	453.61^g^ ± 1.99	58.31^b^ ± 0.63	98.62^e^ ± 0.93	82.41^e^ ± 0.47	450.71^i^ ± 1.56	9.66^a^ ± 0.69	2.90^d^ ± 0.32
10,612	12.81^h^ ± 0.24	23.31^b^ ± 0.77	55.07^e^ ± 0.66	481.32^b^ ± 3.15	55.12^d^ ± 0.47	99.23^d^ ± 1.02	77.31^h^ ± 0.42	527.92^f^ ± 1.24	3.27^e^ ± 0.39	3.27^c^ ± 0.44
11,612	19.51^b^ ± 0.49	23.32^b^ ± 0.79	47.86^h^ ± 0.57	479.31^c^ ± 2.66	47.91^g^ ± 0.55	100.12^c^ ± 1.01	77.31^h^ ± 0.52	717.93^e^ ± 1.43	0.09^f^ ± 0.01	0.41^e^ ± 0.04

*Values are means ± SD of three replicates. Mean values in a column with different superscripts (a, b, c) are significantly different at level p ≤ 0.05. CHO, carbohydrates. Brand No.: 1st digit (1, 2, 3, ……., 5) is a serial number; digits 012 denote age 0–12 months. Brand No. 1st digit (1, 2, 3, ……., 10, 11) is a serial number; digits 612 denote age 6–12 months*.

**Table 3:** Chemical composition (%) of milk formula for infants with age ranging from 1 to 3 years.

**Brand**
**No**.	**Protein (%)**	**Fat (%)**	**CHO (%)**	**Energy (Kcal/100 g)**	**Lactose (%)**	**Total Solids (%)**	**Total non-fat solids (%)**	**Ca (mg/100 g)**	**Fe (mg/100 g)**	**Zn (mg/100 g)**
113	16.82^b^ ± 0.71	19.71^c^ ± 0.46	57.86^a^ ± 0.59	476.11^b^ ± 2.33	57.91^a^ ± 1.24	102.31^a^ ± 1.15	84.41^a^ ± 0.94	641.71^d^ ± 3.24	7.06^c^ ± 0.67	3.21^c^ ± 0.74
213	16.12^b^ ± 0.58	17.53^d^ ± 0.53	56.22^b^ ± 0.71	447.12^d^ ± 3.24	56.33^b^ ± 1.24	97.82^b^ ± 0.98	82.03^b^ ± 0.57	739.73^b^ ± 2.71	10.95^a^ ± 0.49	6.81^a^ ± 0.54
313	13.12^c^ ± 0.48	22.91^a^ ± 0.66	58.16^a^ ± 0.77	491.31^a^ ± 2.89	58.12^a^ ± 1.24	101.92^a^ ± 1.04	80.71^c^ ± 0.81	698.22^c^ ± 4.12	8.55^b^ ± 0.77	5.70^b^ ± 0.61
413	18.71^a^ ± 0.39	21.32^b^ ± 0.68	46.75^c^ ± 0.53	453.73^c^ ± 1.95	46.82^c^ ± 1.24	96.33^c^ ± 1.17	75.32^d^ ± 0.39	910.72^a^ ± 4.62	0.07^d^ ± 0.02	0.43^d^ ± 0.01

*Values are means ± SD of three replicates. Mean values in a column with different superscripts (a, b, c) are significantly different at level p ≤ 0.05. CHO, carbohydrates. Brand No.: 1^st^ digit (1, 2, ……., 4) is a serial number; digits 13 denote age 1–3 months*.

During infancy, a high quantity of protein is mandatory as it is essential for normal growth of the whole body and tissue repair of the infants. The current study discovered that protein contents among most examined formulas had differed significantly. The majority of the formula's protein content was within the range stated by Codex Alimentarius (12. 0–20%) (8). Moreover, it was observed that the maximum content of the protein decreased with age, which agrees with Ballard and Morrow (21), who reported that the protein levels were reduced in human breastmilk over the first 4 to 6 weeks or more time of life irrespective of the timing of delivery.

Fat content was ranged from 26.92% for Brand 206 to 18.53% for Brand 406 for infants of age 0–6 months (Table 1), while, for those of age 0–12 months, fat content was ranged from 26.31 to 18.41% (Table 2) and, for those of age 6–12 months, ranged from 24.92 to 17.31%. However, for those of age 1–3 years, the contents of fat significantly (*p* ≤ 0.05) varied from 22.91 to 17.53% (Table 3). Fat, besides energy, provides fat-soluble vitamins and is necessary for the effective absorption of carotenoids, fat-soluble vitamins, and cholesterol (22). The fat content for all formulas is lower than the range reported by Codex Alimentarius (29.3–40.%) (8).

Carbohydrates content was ranged from 60.64% for Brand 1,006 to 48.76% for Brand 706 for formulas prepared for an infant of age 0–6 months (Table 1), while, for those of age 0–12 months, carbohydrates content was ranged from 63.37 to 44.97% (Table 2) and, for those of age 6–12 months, was ranged from 63.27 to 46.03%. Moreover, for those of age 1–3 years, the contents of carbohydrates significantly (*p* ≤ 0.05) varied from 58.16 to 46.75% (–). A higher carbohydrate level in milk had a significant role in infant nutrition. Both digestible and indigestible carbohydrates were available in human milk, while infant formulas contained only digestible ones (23). The carbohydrate content of the present formulas was lower than the range reported by Codex Alimentarius (60.0–93.3%) (8). A significant (*p* ≤ 0.05) variation in total energy was observed between the formulas for all ages.

The total energy was ranged from 517.44 Kcal/100 g for Brand 506 to 457.01 Kcal/100 g for Brand 706 for formulas prepared for an infant of age 0–6 months (Table 1), while, for those of age 0–12 months, the total energy was ranged from 544.71 to 424.43 Kcal/100 g and, for those of age 6–12 months, was ranged from 497.31 to 449.52 Kcal/100 g (Table 2). Moreover, for those of age 1–3 years, the total energy significantly (*p* ≤ 0.05) varied from 491.31 to 447.12 Kcal/100 g (Table 3). The values reported for the formulas were higher than those reported by Schoen et al. (24).

### Lactose, Total Solids, and Total Non-Fat Solids of Infant Formulas

According to the data provided, a significant (*p* ≤ 0.05) variation was observed in lactose content among the formula under investigation. As shown in Table 1, the formula for infants of age 0–6 months contained 60.72 and 48.83%, with Brand 1,006 having a higher amount than other formulas. For infants of age 6–12 months, lactose ranged from 63.32 to 46.13%, and, for those of age 0–12 months, was ranged from 63.42 to 45.01% (Table 2). However, for those aged 1–3 years, lactose ranged from 58.12 to 46.82% (Table 3). It has been reported that lactose, the main carbohydrate found in humans, cow, and goat milk, is the foremost and dominant carbohydrate in the infant formula (25). It is also the most abundant component of the infant formula, accounts for 35–50% of the bulk formation, and serves as an important source of energy (25). Moreover, they reported that lactose must contribute to 18% of infant formula. Guerra-Hernández et al. (26) reported similar values for infant formula.

Total solids and total non-fat solids were significantly (*p* ≤ 0.05) varied between the formulas. Infants of age 0–6 months were found to be ranged from 104.31 to 96.52% and from 81.61 to 72.01% for total solids and total non-fat solids, respectively (Table 1). The formula for infants with age ranged from 6–12 months, total solids and total non-fat solids, respectively, ranged from 102 to 97.41% and from 86.61 to 76.93%, while, for those of age 0–12 months, ranged from 109.62 to 88.81% and from 86.51 to 65.71%, respectively (Table 2). For infants aged 1–3 years, total solids ranged from 102.31 to 96.33%, while total non-fat solids ranged from 84.41 to 75.32% (Table 3). Masum et al. (9) reported values for total solids similar to most of the formulas under investigation.

### Calcium, Iron, and Zinc of Infant Formulas

The current investigation showed that calcium contents varied significantly (*p* ≤ 0.05) between milk formulas and ranged from 754.12 to 303.42 mg/100 gm, with a higher significant (*p* ≤ 0.05) value obtained for Brand 706 for infants of age 0–6 months (Table 1). As shown in Table 2, Brand 7,612 prepared for those of age 6–12 months recorded a significantly (*p* ≤ .05) higher value of Ca (943.64 mg/100 gm) than Brand 4,612, which recorded the least value (278.43 mg/100 gm). Moreover, formulas for those of age 0–12 months contained Ca in a range of 664.93–371.81 mg/100 gm. The calcium content of formulas for infants of age 1–3 years ranged from 910.72 to 641–71 mg/100 gm (Table 3). A study conducted by Kotb et al. (27) on the chemical composition of milk formulas sold in Egypt showed similar results. Another study conducted by Molska et al. (28) reported that the investigated formulas contained significantly (*p* ≤ 0.05) higher Ca than the recommended intake. They concluded that, despite the lesser bioavailability of calcium in the formulas, there might be a risk of exceeding the daily intake limits, which may cause kidney difficulties and impair the absorption of iron, magnesium, and zinc (29). The minimum calcium content in infant formulas should be 333.1 ppm (8).

A significant (*p* ≤ 0.05) variation was observed in Fe content between the formulas. The current investigation showed that iron contents ranged from 11.73 to.10 mg/100 gm for infants aged 0–6 months (Table 1). For infants of age 6–12 months, Fe was in the range of 9.77–0.07 mg/100 gm, while, for those of age 0–12 months, it was in the range of 11.97-.03 mg/100 gm (Table 2). As shown in Table 3, for those of age 1–3 years, Brand 213 had a significantly (*p* ≤ 0.05) higher content of Fe (10.95 mg/100 gm) than Brand 413, which contained the least amount of Fe (.07 mg/100 gm). The highest iron content in some formulas could be attributed to fortification with iron. Iwegbuea et al. (30) studied the trace element composition of infant formulas commercially accessible in the Nigerian market. Saracoglu et al. (31) analyzed the milk formulas in Turkey, and the obtained data are comparable to the present study. The minimum iron content in milk formulas should not be <0.45 mg/100 kcal (8). For infants aged 4–5 months, the tissue requirement for iron increases, so formulas should be supplemented with iron (32). A remarkable shortage in the iron quantity of infants may lead to impaired cell-mediated immunity, while an excessive amount of the same may lead to severe liver damage (3).

Zinc content varied significantly (*p* ≤ 0.05) between the formulas and was found to range from 13.97 to.42 mg/100 gm for infants aged 0–6 months (Table 1). A range of 5.50 to −0.41 mg/100 gm was obtained in formulas for infants of age 6–12 months, while, for those of age 0−12 months, it ranged from 3.99 to 0.40 mg/100 gm (Table 2). However, for those of age 1–3 years, it ranged from 6.81 to 0.41 mg/100 gm (Table 3). Iwegbuea et al. (30) analyzed the commercial milk formulas in the Nigerian market for trace element composition and reported lower values than the present study in Zn content. Codex Alimentarius assign minimum zinc content of 5.0 mg/100 kcal in infant milk formulas. Zinc is required to synthesize protein and nucleic acid metabolism (33). A recommended daily intake of Zn is 5.0 mg/day for infants. The concentration of Zn in the mother milk decreased with the time of lactation, and the percent reduction reached 20% after 3 months (28). Excessive zinc intake can cause microcytic anemia and reduce the iron concentration in the infant body (34). Zinc competes with Mg (magnesium) at the absorption level in the intestines and the structural parts of the bone (28).

## Conclusion

According to the cited literature and the Codex regulations for infant food, the majority of the investigated milk formulas' carbohydrates and fat contents were lower than the range. The protein and mineral contents, on the other hand, were within the Codex Alimentarius range. Infant nutrition may be affected by the lower carbohydrate and fat content. Further studies are needed to evaluate milk formulas on larger quantities of samples and a variety of milk brands to guarantee the accuracy of the contents advertised by the producer. All health organizations are concerned about the health of infants. As a result, milk formulas that have replaced human milk should be carefully considered from a nutritional standpoint.

## Data Availability Statement

The original contributions presented in the study are included in the article/supplementary material, further inquiries can be directed to the corresponding author/s.

## Author Contributions

NA, TA, and JA contributed to preparing the experimental results and the manuscript. ZA and SW helped in writing and editing the manuscript. All authors contributed to the article and approved the submitted version.

## Funding

This research was supported by Princess Nourah bint Abdulrahman University Researchers Supporting Project Number (PNURSP2022R34), Princess Nourah bint Abdulrahman University, Riyadh, Saudi Arabia.

## Conflict of Interest

The authors declare that the research was conducted in the absence of any commercial or financial relationships that could be construed as a potential conflict of interest.

## Publisher's Note

All claims expressed in this article are solely those of the authors and do not necessarily represent those of their affiliated organizations, or those of the publisher, the editors and the reviewers. Any product that may be evaluated in this article, or claim that may be made by its manufacturer, is not guaranteed or endorsed by the publisher.
